# SELF-COMPASSION, DAILY STRESS APPRAISALS, AND ODDS OF ELDER ABUSE AND NEGLECT IN DEMENTIA FAMILY CAREGIVING

**DOI:** 10.1093/geroni/igad104.2019

**Published:** 2023-12-21

**Authors:** Carolyn Pickering, Mustafa Yildiz, Maria Yefimova

**Affiliations:** University of Alabama at Birmingham, Birmingham, Alabama, United States; University of Alabama at Birmingham, Birmingham, Alabama, United States; UCSF Center for Nursing Excellence & Innovation, San Franscico, California, United States

## Abstract

Stress related to behavioral symptoms is often found to be a risk factor for elder abuse and neglect in dementia family caregiving. The stress-process model suggests that stressful events alone do not determine caregiving outcomes, rather, they are impacted by personal factors which influence the response to stress. Self-compassion represents how one responds to themselves in moments of distress, with compassionate self-responding consisting of treating oneself with self-kindness (vs. judgement), feelings of common humanity (vs. isolation), and mindful awareness (vs. overidentification). Self-compassion is a modifiable intervention target with efficacious interventions available. We hypothesize that self-compassion can buffer the effects of daily stress on a caregiver’s likelihood of using abusive and neglectful behaviors. To test this hypothesis, a generalized linear mixed model with days (n=9513) nested in dementia family caregivers (N=453) was used. Self-compassion was measured at baseline with the Self-Compassion Scale – Short Form, and daily stress appraisal of behavioral symptoms and occurrences of elder abuse and neglect were measured on 21 sequential daily diary surveys. Having higher self-compassion reduced the likelihood of a caregiver using abusive and neglectful behaviors on a given day by 40% (p=0.001, CI 0.45-0.80). Daily stress appraisal of behavioral symptoms significantly increased the odds of abuse and neglect (OR 1.05, CI 1.02-1.09, p=0.002), while the interaction term showed self-compassion significantly lowered the effect of daily stress (OR 1.01, CI 1-1.02, p=0.029). Given the large effect size, self-compassion should be a priority area for future interventions to reduce elder abuse and neglect in dementia family caregiving.

